# Bone loss, low height, and low weight in different populations and district: a meta-analysis between vegans and non-vegans

**DOI:** 10.29219/fnr.v64.3315

**Published:** 2020-09-11

**Authors:** Jianfeng Li, Ruiyun Zhou, Wei Huang, Jianjun Wang

**Affiliations:** 1 Department of Spine and Osteology, Zhuhai People’s Hospital (Zhuhai Hospital Affiliated with Jinan University), Zhuhai, Guangdong Province, China; 2 Zhuhai Medical Emergency Center, Pre-Hospital Emergency Training Base of Mid-southern China, Zhuhai, Guangdong Province, China

**Keywords:** bone mineral density, height, weight, vegan, meta-analysis

## Abstract

**Objective:**

The aim of this study was to- conduct a meta-analysis of the association of bone mineral density, height, and weight in different populations between vegans and non-vegans.

**Methods:**

Based on a search of PubMed, Web of Science, MEDLINE, the Cochrane Library, the Wanfang database, and the CNKI database, 14 relevant publications were collected by two researchers. Review Manager 5.3 and Stata 12.0 software were used for data analysis.

**Results:**

The following results were observed in this study: 1) the density of lumbar vertebrae was higher in vegans than in non-vegans (mean difference: -0.05, 95% CI: -0.09 to -0.01, *P* = 0.01); 2) hip bone density was higher in non-vegans than in vegans (mean difference: -0.08, 95% CI: -0.14 to -0.02, *P* = 0.008); 3) weight was higher in non-vegans than in vegans (mean difference: -2.21, 95% CI: -4.05 to -0.37, *P* = 0.02); and 4) height was higher in non-vegans than in vegans (mean difference: -1.87, 95% CI: -2.52 to -1.22, *P* < 0.00001).

**Conclusion:**

Our study suggests that a vegetarian lifestyle may contribute to bone loss, low height, and low weight based on existing evidence.

## Popular scientific summary

- There are three traditional dietary modes in Europe and the United States (Western, Mediterranean and vegetarian diets). Western dietary patterns are common in the United States, Canada and some countries in northern Europe. The carbohydrate energy supply ratio is only 25% and the fat energy supply is high, reaching 35% to 45%, in which the saturated fatty acid accounts for 18% in Western diets. In addition, the Western diet is high in protein and low in dietary fiber. Western dietary patterns are the main causes of metabolic diseases, such as obesity and type II diabetes in the United States [1].
- The Mediterranean diet (MD), as the name implies, is the diet of inhabitants of the Mediterranean, such as Italy and Greece. The MD is rich in whole grains, beans, fruits, vegetables, nuts and other plant-based foods. Olive oil is the main fat source in the MD, with moderate intake of fish and poultry and a relatively small intake of livestock, sweets and dairy products. Most adults drink wine. The energy supply ratio of the MD is 25% to 35%, in which the saturated fatty acid accounts for only 7% to 8% [2]. In addition, the MD is rich in dietary fiber and a low glycemic index, which can help prevent type II diabetes and cardiovascular disease [3].
- The vegetarian model can be divided into vegan, vegetarian and semi-vegetarian types, depending on the food that is eaten. Veganism refers to the elimination of all animal-based foods in the diet and a substantial intake of fruits, vegetables, beans, nuts and soy protein. The fat supply ratio is appropriate, but an omnivore consumes more polyunsaturated fatty acids than a vegan. Vegetarian diets are similar to those described above, but can include eggs and milk. Semi-vegetarian diets include red meat, poultry and fish, but not more than one time per week [4]. Studies have shown that vegetarians are at risk for a variety of important nutrient deficiencies, including protein, calcium, iron, iodine, vitamin D and vitamin B12, suggesting that a vegetarian diet has a negative impact on bone growth and development [5,6]. For this reason, the current study increased the sample size and improved the test efficiency through a meta-analysis to obtain more authentic and reliable analysis results, which helped to clarify whether a vegetarian diet has negative effects on bone growth and development, and provided evidence-based medicine for clinical diagnosis and treatment.

There are three traditional dietary modes in Europe and the United States (Western, Mediterranean, and vegetarian diets). Western dietary patterns are common in the United States, Canada, and some countries in northern Europe. The carbohydrate energy supply ratio is only 25% and the fat energy supply is high, reaching 35–45%, in which saturated fatty acid accounts for 18% in Western diets. In addition, the Western diet is high in protein and low in dietary fiber. Western dietary patterns are the main causes of metabolic diseases, such as obesity and type 2 diabetes, in the United States (1).

The Mediterranean diet (MD), as the name implies, is the diet of inhabitants of the Mediterranean, such as Italy and Greece. The MD is rich in whole grains, beans, fruits, vegetables, nuts, and other plant-based foods. Olive oil is the main fat source in the MD, with moderate intake of fish and poultry and a relatively small intake of livestock, sweets, and dairy products. Most adults drink wine. The energy supply ratio of the MD is 25–35%, in which the saturated fatty acid accounts for only 7–8% (2). In addition, the MD is rich in dietary fiber and has a low glycemic index, which can help prevent type II diabetes and cardiovascular disease (3).

The vegetarian model can be divided into vegan, vegetarian, and semi-vegetarian types, depending on the food that is eaten. Veganism refers to the elimination of all animal-based foods in the diet and a substantial intake of fruits, vegetables, beans, nuts, and soy protein. The fat supply ratio is appropriate, but an omnivore consumes more polyunsaturated fatty acids than a vegan. Vegetarian diets are similar to those described above, but can include eggs and milk. Semi-vegetarian diets include red meat, poultry, and fish, but not more than one time per week (4). Studies have shown that vegetarians are at risk for a variety of important nutrient deficiencies, including protein, calcium, iron, iodine, vitamin D, and vitamin B12, suggesting that a vegetarian diet has a negative impact on bone growth and development (5, 6). For this reason, the current study increased the sample size and improved the test efficiency through a meta-analysis to obtain more authentic and reliable analysis results, which helped clarify whether a vegetarian diet has negative effects on bone growth and development, and provided evidence-based medicine for clinical diagnosis and treatment.

## Materials and methods

### Study selection

By searching PubMed, Web of Science, MEDLINE, the Cochrane Library, the Wanfang database, and the CNKI database for articles published before January 2018, a total of 14 relevant studies were identified. The following keywords were searched: “bone mineral density or bone loss or osteopenia or osteoporosis,” “vegans or vegetarians or veganism or lacto-ovo-vegetarian,” and(or) “non-vegans or omnivores.” To identify titles and abstracts of the relevant literature, reference lists of studies were checked manually. The retrieval time was from January 1991 to March 2018.

### Inclusion and exclusion

All studies included in the meta-analysis met the following criteria: 1) assessment of the bone mineral density (BMD), height, and weight between vegans and non-vegans; 2) case-controlled trial and the controls had no malignant disease; 3) detailed data of studies must be completely provided in the experimental and control groups directly or indirectly; and 4) all studies had similar research methods and purposes. The exclusion criteria were as follows: 1) repeat studies; 2) no control group and research samples <10; 3) incomplete description of data or unclear sample data; 4) animal experimental research; and 5) articles composed of reviews, abstracts, discussions, letters, annotations, and case reports.

### Data extraction

Data were extracted by two reviewers. Extraction of literature included the first author, publication date, country, methods, and basic characteristics of the patients (including age, gender, and quantity).

### Quality assessment

The STROBE scoring system was used to evaluate the quality of the study (7). There are 22 scoring items in the STROBE scoring system. A score of 0–17.5 is low quality, 17.5–35 is medium quality, and 35–44 is high quality. All studies included were of medium- and high-quality research.

### Data analysis

Stata 12.0 and Review Manager 5.3 software were used for the meta-analysis, as follows: 1) the combined BMD, height, and weight and 95% confidence intervals were calculated; 2) Funnel plot analysis, Begg’s test, and Egger’s test were used for publication bias; 3) heterogeneity between studies was evaluated using a χ^2^-based *Q* test and *I*^2^ test (*I*^2^ = 75–100%, extreme heterogeneity; *I*^2^ = 50–75%, large heterogeneity; *I*^2^ = 25–50%, moderate heterogeneity; *I*^2^ < 25%, no heterogeneity); if there was heterogeneity (*I*^2^ > 50%), the random-effect model was adopted, otherwise the fixed effect model was used; 4) sensitivity analysis was performed by removing one study at a time to compare the difference of pooled effects before and after deleting the study; if the pooled results were reserved after removing the study, it indicated that the results was unstable; 5) subgroup analysis was based on age and populations; and 6) *P* < 0.05 was considered statistically significant.

## Results

### Characteristics of studies

**Fig. 1:**
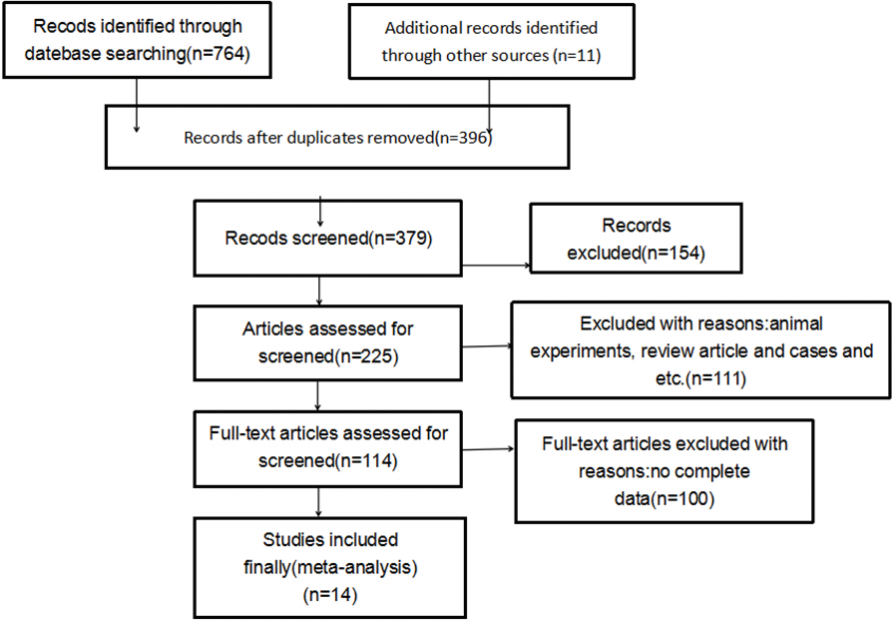
Literature search flow diagram.

Based on the above retrieval methods, 775 relevant studies were selected. After reading the titles and abstracts, and reviewing the full text, 761 studies were excluded. Fourteen case–control studies involving 1,763 subjects were selected for the meta-analysis, as shown in Fig. 1. The main characteristics of the eligible studies are presented in Table 1.

**Table 1:** Characteristics of the studies included in the meta-analysis

First author	Year	Country/district	Number	Age (years)	Female (%)	Intervention	Vegan duration	Evaluated	STROBE score
			Cases/controls	Cases/controls	Cases/controls				
Hunt (8)	1989	USA	144/146	66.6 ± 10.0/65.7 ± 8.5	100%/100%	Vegetarian	Ages from 47 to 98	BMD, Height, Weight	22
Chiu (9)	1997	Taiwan	171/187	63.97 ± 11.47/59.63 ± 8.00	100%/100%	Buddhist nuns or Buddhist followers	Long-term	BMD, Height, Weight	23
Lau (10)	1998	Hong Kong	76/109	79.1 ± 5.2/77.0 ± 3.8	100%/NA	Vegetarian	Over 30 years	BMD, Height, Weight	22
Barr (11)	1998	Britain	23/22	26.6 ± 4.3/27.9 ± 5.9	100%/100%	Premenopausal Vegetarian	Over 2 years	BMD, Height, Weight	23
Siani (12)	2003	Italy	20/10	34.78 ± 15.07/38.40 ± 7.77	NA	Vegetarians	Entire lives	BMD, Height, Weight	24
Fontana (13)	2005	USA	18/18	56.5 ± 13.1/53.2 ± 4.1	NA	Vegetarian diet	Mean of 3.6 years	BMD, Height, Weight	25
Hopham (14)	2009	Vietnam	105/105	62 ± 10/62 ± 10	100%/100%	Buddhist nuns	No meat or seafood	BMD, Height, Weight	22
Sambol (15)	2009	Croatia	20/50	34.9 ± 9.2/36.2 ± 9.2	60%/68%	Vegetarian diet	NA	BMD	26
Chen (16)	2010	China	62/60	30.81 ± 5.18/31.4 ± 4.88	NA	Buddhist followers	Over 5 years	BMD, Height, Weight	25
Hopham (17)	2012	Vietnam	88/93	61 ± 9.2	100%/100%	Buddhist nuns	Strictly vegans	BMD, Height, Weight	27
Knurick (18)	2015	USA	18/19	33.9 ± 8.6/27.2 ± 6.7	64%/70%	Vegetarian	NA	BMD, Height, Weight	22
Elham (19)	2018	Canada	31/32	12.0 ± 1.8	100%/100%	Vegetarian	NA	BMD	22
Jadwiga (20)	2018	Poland	70/60	6.56 ± 1.8/6.88 ± 1.5	NA	Vegetarian diet	From birth	BMD, Height, Weight	23
Ambroszkiewicz (21)	2018	Poland	53/53	5.3–9.0/5.4–8.9	NA	Vegetarian diet	From birth	BMD, Height, Weight	24

### Meta-analysis results

Comparison of lumbar vertebrae density between vegetarians and non-vegetarians

Fourteen articles (7–21) provided data on 1,763 cases, including 799 vegetarians and 964 non-vegetarians.

1. The heterogeneity test showed statistically significant differences (χ^2^ test = 236.01, *P* < 0.00001, *I*^2^ = 94%). Therefore, a random-effect model analysis was used to show that lumbar vertebral density in the non-vegetarian group was higher than that in the vegetarian group (Fig. 2; mean difference: -0.05, 95% CI: -0.09 to -0.01, *P* = 0.01).
2. Sensitivity analysis revealed that the Chen study (16) had a greater impact on the stability of the conclusion (Fig. 2c). After removing the Chen study (16), the lumbar vertebral density of the non-vegetarian group was higher than the vegetarian group (mean difference: -0.03, 95% CI: -0.05 to -0.01, *P* = 0.0006). Heterogeneity analysis was carried out by removing small sample size, which influenced sensitivity. After removing Chen (16), Fontana (13), Knurick (18), Barr (11), Siani (12), and Sambol (15) studies, the heterogeneity was low (χ^2^ = 8.84, *P* = 0.26, *I*^2^ = 21%), and the lumbar vertebral density of the non-vegetarian group was higher than the vegetarian group (mean difference: -0.03, 95% CI:, *P* < 0.00001).
3. According to the subgroup analysis, compared with the age group, the lumbar vertebrae density of the non-vegetarian diet group <25 years or >50 years of age was higher than that of the vegetarian diet group (mean difference: -0.04, 95% CI: -0.05 to -0.03, *P* < 0.00001; mean difference: -0.04, 95% CI: -0.07 to -0.01, *P* = 0.02; subgroup difference: χ^2^ = 0.01, *P* = 0.99, *I*^2^ = 0%; Fig. 2a), and compared with the regional group, the density of lumbar vertebrae in the non-vegetarian group was higher than that in the vegetarian group (mean difference: -0.05, 95% CI: -0.09 to -0.01, *P* = 0.01; subgroup difference: χ^2^ = 1.86, *P* = 0.39, *I*^2^ = 0%; Fig. 2b).

**Fig. 2a:**
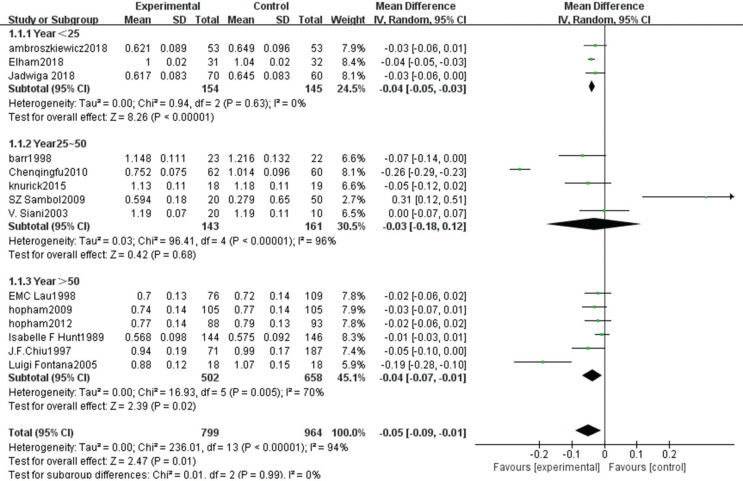
Meta-analysis of bone mineral density in spine between different age groups.

**Fig. 2b:**
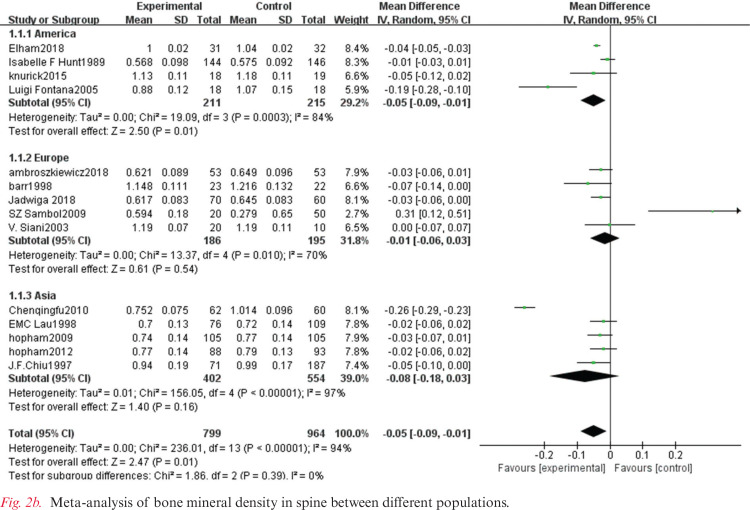
Meta-analysis of bone mineral density in spine between different populations.

**Fig. 2c:**
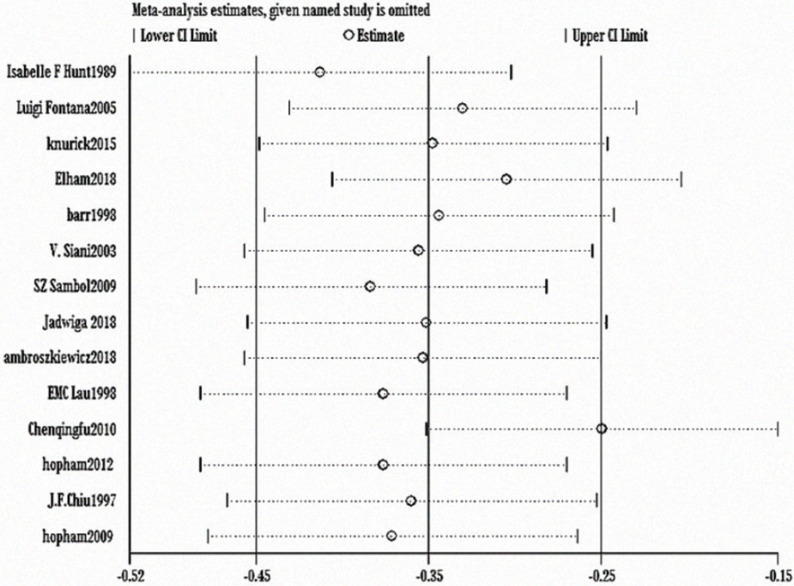
Sensitivity analysis of lumbar vertebrae density.

Comparison of hip bone density between vegetarians and non-vegetarians

Eight articles (9–10, 13–14, 16–19) provided data on 1,072 cases, including 469 vegetarians and 623 non-vegetarians.

1. The heterogeneity test showed statistical differences (χ^2^ = 244.95, *P* < 0.00001, *I*^2^ = 97%). Therefore, a random-effect model analysis was used to show that hip bone density was higher in the non-vegetarian group than in the vegetarian group (Fig. 3b; mean difference: -0.08, 95% CI: -0.14 to -0.02, *P* = 0.008).
2. Sensitivity analysis revealed that the Chen study (16) had a greater impact on the stability of the conclusion. After removing this study, significantly higher hip bone density in the non-vegetarian group than that in the vegetarian group was maintained as the confidence intervals of mean difference among included studies were closer (Fig. 3c; mean difference: -0.04, 95% CI: -0.05 to -0.03, *P* < 0.0001). Heterogeneity analysis was carried out by removing small sample size and influencing sensitivity. After removing the Chen (16), Fontana (13), and Knurick (18) studies, the heterogeneity was low (χ^2^ = 6.24, *P* = 0.18, *I*^2^ = 36%) and the BMD of the hip in the non-vegetarian group was higher than that in the vegetarian group (mean difference: -0.04, 95% CI: -0.05 to -0.03, *P* < 0.00001).
3. According to subgroup analysis, compared with the age group, hip bone density in the non-vegetarian group was higher than that in the vegetarian group (mean difference: -0.04, 95% CI: -0.05 to -0.03, *P* < 0.00001; mean difference: -0.04, 95% CI: -0.08 to -0.01, *P* = 0.02; subgroup difference: χ^2^ = 0.03, *P* = 0.87, *I*^2^ = 0%; Fig. 3a), and based on a regional comparison, hip bone density was greater in the non-vegetarian group than the vegetarian group (mean difference: -0.06, 95% CI: -0.10 to -0.02, *P* = 0.007; subgroup difference: χ^2^ = 0.25, *P* = 0.61, *I*^2^ = 0%; Fig. 3b).

**Fig. 3a:**
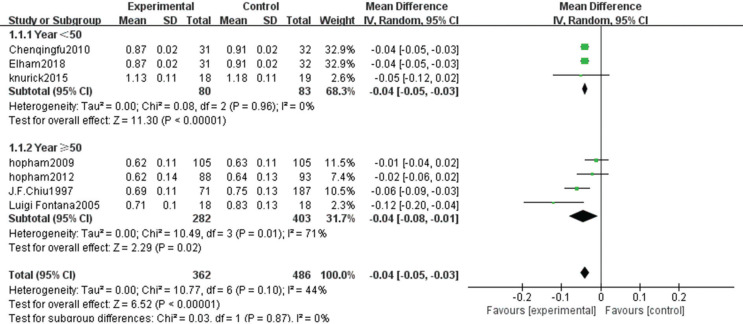
Meta-analysis of bone mineral density in hip between different age groups.

**Fig. 3b:**
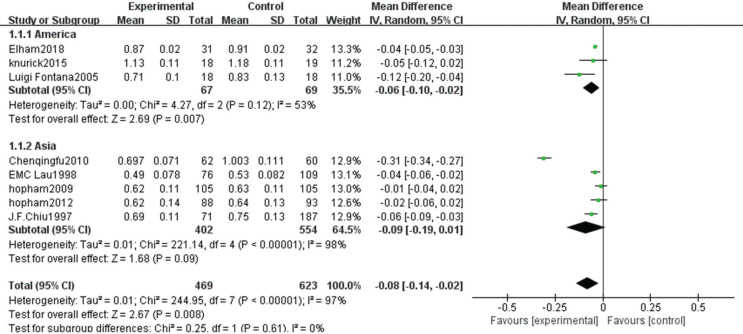
Meta-analysis of bone mineral density in hip between different populations.

**Fig. 3c:**
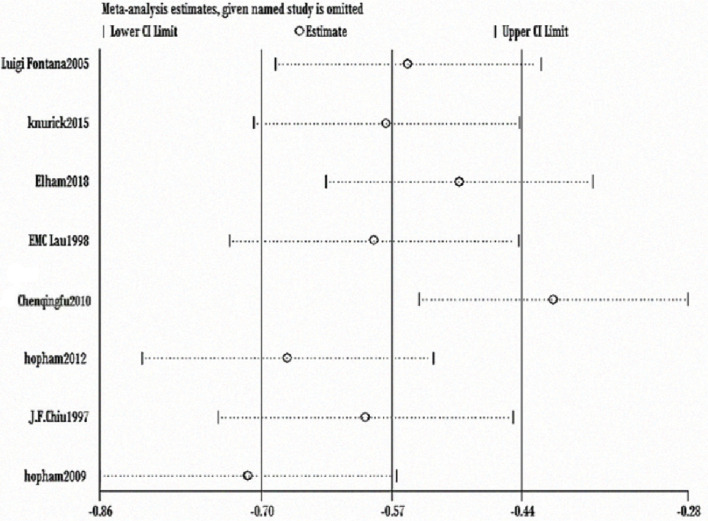
Sensitivity analysis of hip bone density.

Weight comparison between vegetarians and non-vegetarians

Twelve articles (7–14, 16–19, 21) provided data on 1,630 cases, including 748 vegetarians and 882 non-vegetarians.

1. The heterogeneity test showed statistical evidence of differences (χ^2^ = 39.67, *P* < 0.00001, *I*^2^ = 75%). Therefore, a random-effect model analysis was used to show that the weight of the non-vegetarian group was greater than that of the vegetarian group (Fig. 4; mean difference: -2.21, 95% CI: -4.05 to -0.37, *P* = 0.02).
2. Sensitivity analysis revealed that the Hunt study (8) had a significant impact on the strength of the conclusion. After removing this study, the weight of the non-vegetarian group was still greater than the vegetarian group (mean difference: -1.55, 95% CI: -2.45 to -0.65, *P* = 0.0008). Heterogeneity analysis was carried out by removing small sample size and influencing sensitivity. After removing the Hunt (8), Fontana (13), Knurick (18), and Siani (12) studies, the heterogeneity was relatively low (χ^2^ = 1.86, *P* = 0.93, *I*^2^ = 0%) and the weight of the non-vegetarian group was still greater than that of the vegetarian group (mean difference: -0.98, 95% CI: -1.91 to -0.04, *P* = 0.04).
3. According to subgroup analysis, compared with the age group, the weight of the non-vegetarian group 25–50 years of age was greater than the vegetarian group (mean difference: -2.84, 95% CI: -5.13 to -0.55, *P* = 0.02; subgroup difference: χ^2^ = 1.37, *P* = 0.51, *I*^2^ = 0%; Fig. 4a), and based on regional comparison, the weight of the non-vegetarian group was equal to the vegetarian group (subgroup difference: χ^2^ = 1.95, *P* = 0.38, *I*^2^ = 0%; Fig. 4b).

Comparison of height between vegetarians and non-vegetarians

Twelve articles (7–14, 16–19, 21) provided data on 1,630 cases, including 748 vegetarians and 882 non-vegetarians.

1. A heterogeneity test showed no statistical differences (χ^2^ = 8.53, *P* = 0.67, *I*^2^ = 0%). Therefore, a fixed-effect model analysis was adopted, which indicated that the height of the non-vegetarian group was greater than that of the vegetarian group (Fig. 5; mean difference: -1.87, 95% CI: -2.52 to -1.22, *P* < 0.00001).
2. Sensitivity analysis revealed that the Chiu study (9) had a greater impact on the strength of the conclusion. After removing this study, the height of the non-vegetarian group was still greater than that of the vegetarian group (mean difference: -1.59, 95% CI: -2.32 to -0.85, *P* < 0.0001). Heterogeneity analysis was carried out by removing small sample size and influencing sensitivity. After removing the Chiu (9), Fontana (13), Knurick (18), Barr (11), and Siani (12) studies, the heterogeneity was relatively low (χ^2^ = 2.19, *P* = 0.90, *I*^2^ = 0%) and height of the non-vegetarian group was greater than that of the vegetarian group (mean difference: -1.70, 95% CI: -2.47 to -0.93, *P* < 0.00001).
3. According to subgroup analysis, 1) based on a comparison of the age group, the height of the non-vegetarian group at 50 years of age was greater than that of the vegetarian group (mean difference: -2.01, 95% CI: -2.72 to -1.31, *P* < 0.00001; subgroup difference: χ^2^ = 2.09, *P* = 0.35, *I*^2^ = 4.2%; Fig. 5a), and 2) based on regional comparison, the height of the non-vegetarian group was greater than that of the vegetarian group (mean difference: -1.70, 95% CI: -3.22 to -0.18, *P* = 0.03) in the American or Asian population (mean difference: -1.95, 95% CI: -2.71 to -1.19, *P* < 0.00001; subgroup difference: χ^2^ = 0.17, *P* = 0.92, *I*^2^ = 0%; Fig. 5b).

**Fig. 4a:**
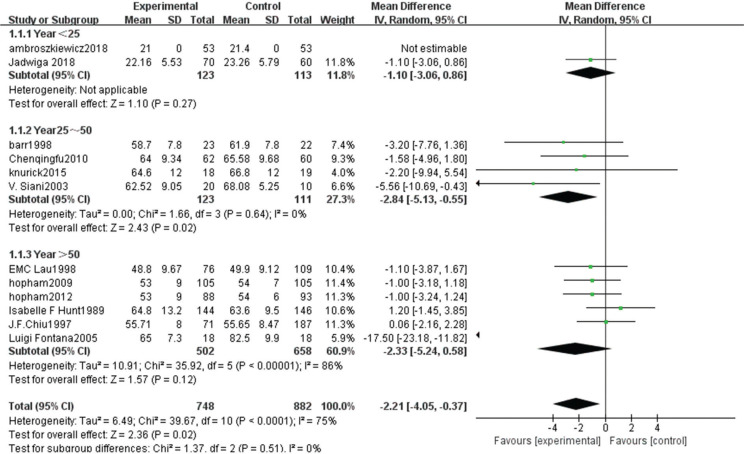
Meta-analysis of weight between different age groups.

**Fig. 4b:**
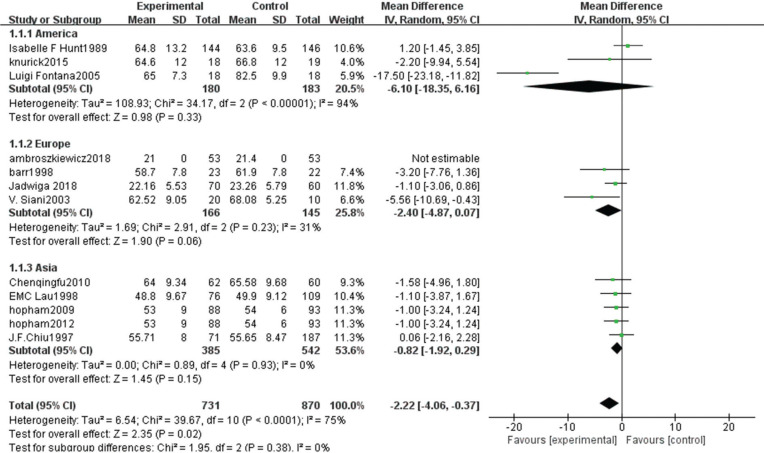
Meta-analysis of weight between different populations.

**Fig. 4c:**
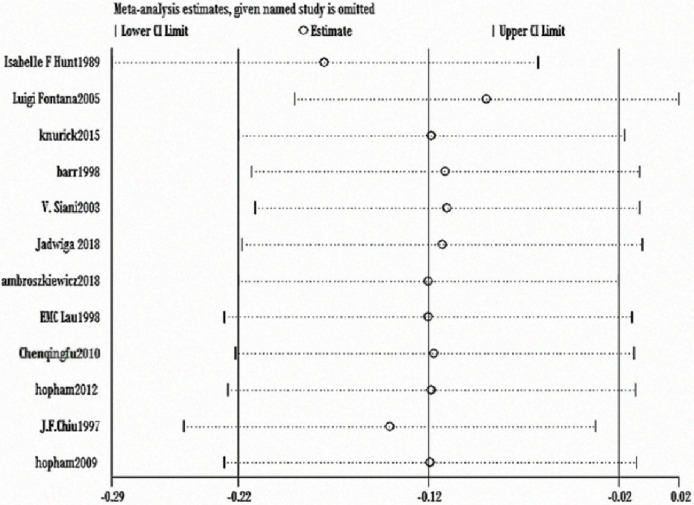
Sensitivity analysis of body weight.

**Fig. 5a:**
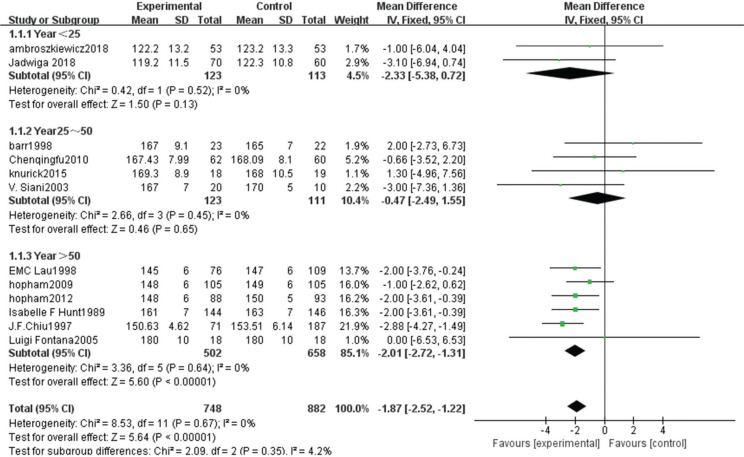
Meta-analysis of height between different age groups.

**Fig. 5b:**
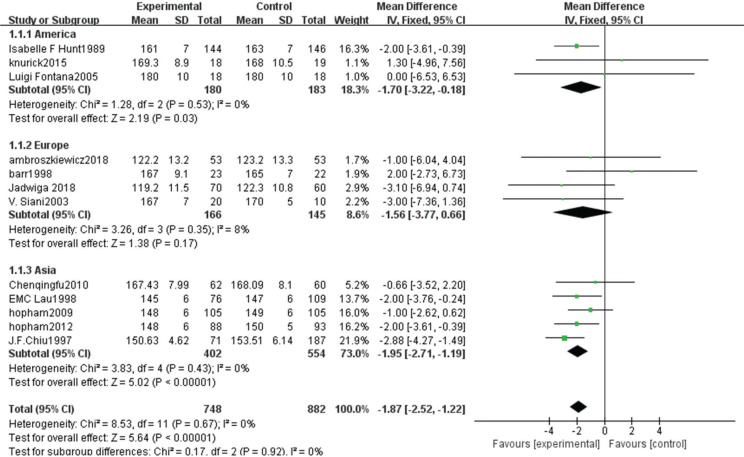
Meta-analysis of height between different populations.

**Fig. 5c:**
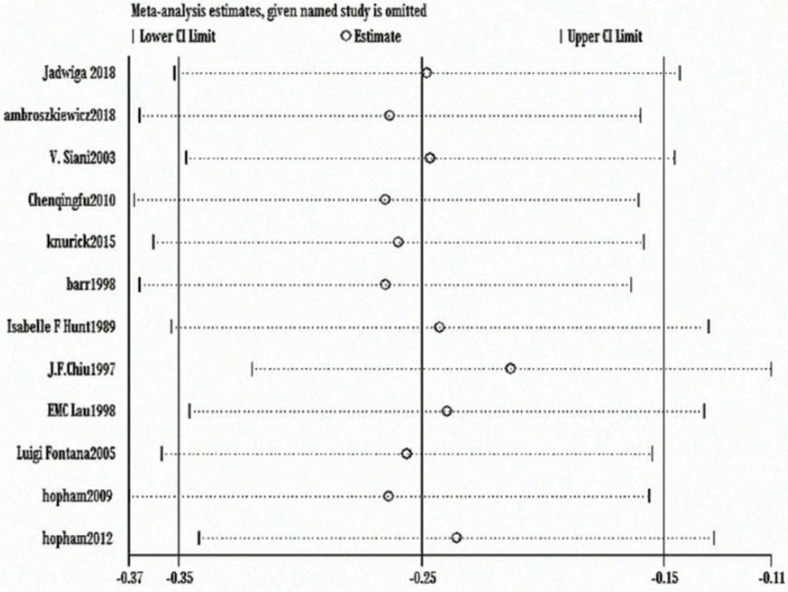
Sensitivity analysis of height.

### Publication bias

**Fig. 6:**
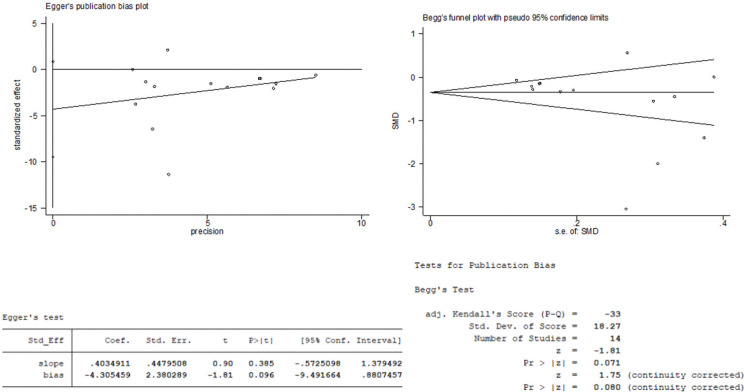
Publication bias test in Egger’s and Begg’s tests.

Egger’s and Begg’s tests were performed to evaluate the publication bias. As shown in Fig. 6, the symmetry of the funnel plots suggested no obvious publication bias in Begg’s test (*P* > 0.05), and the results of Egger’s test suggested no evidence of publication bias (*P* > 0.05).

## Discussion

### Advantages of vegetarian diet

Diet and other lifestyle factors play an important role in the pathogenesis of chronic diseases (22, 23). A healthy lifestyle decreases the prevalence of coronary heart disease by 83% and decreases type II diabetes in women by 91% (24). A vegetarian diet, due to its unique natural ingredients and composition of nutrients, plays an important role in the prevention and treatment of metabolic disorders, including blood lipid disorders, obesity, type II diabetes, coronary heart disease, and metabolic syndrome.

Low-fat vegetarian and traditional diabetic diets result in no significant differences in weight improvement, but a vegetarian diet significantly reduces fasting blood glucose, cholesterol, and triglyceride levels in type II diabetic patients (25, 26). A variety of dietary patterns have shown that the systolic blood pressure level of those who opt for a vegetarian diet is significantly lower (27–29). A meta-analysis by Yokoyama et al. (30) also confirmed this finding. A study involving 55,459 Swedish women conducted by Newby et al. (31) showed that the prevalence of overweight or obesity was 40%, whereas the serum total cholesterol, low-density lipoprotein (LDL), and triglycerides levels were significantly lower in vegetarians (32). Vegan and lacto-vegetarian diets reduce triglycerides and LDL by 10–15%, vegetarian diets reduce triglycerides and LDL by 15–25%, and combined diets (fiber, soy, and nuts added to vegan diets) reduce triglycerides and LDL by 20–35%; for every 1% reduction in LDL, the risk of coronary heart disease is reduced by 1% (33). Therefore, the decreased mortality rate of coronary heart disease in the vegetarian population may be related to the lipid-lowering effect of a vegetarian diet. In addition, vegetarian diets are rich in vitamins and have strong antioxidant and free radical scavenging effects. Oxidative damage is closely related to metabolic syndrome. Vitamin E, together with superoxide dismutase and glutathione peroxidase, make up the antioxidant system in the body and remove free radicals. Vitamin C is transformed into dehydroascorbic acid through oxidation and reduction to remove oxygen- and hydroxyl-free radicals to prevent oxidation-related diseases (34). As the helper factor and precursors of mitochondrial enzymes, B vitamins can protect or activate mitochondrial enzymes, thereby maintaining normal energy metabolism and preventing the occurrence of metabolic diseases (35).

### Disadvantages of a vegetarian diet

From the perspective of nutrition, there are also deficiencies in a vegetarian diet. Firstly, the quality of protein in plant food is poor (except soy protein), and the composition of essential amino acids is incomplete or the quantity of essential amino acids is insufficient. Secondly, a vegetarian diet does not contain vitamin B12 and most essential elements, such as iron and calcium, and zinc is very limited. Oxalic acid, phytic acid, dietary fiber, and the interference by other minerals prevent the absorption of calcium, zinc, and iron, thus causing iron, calcium, zinc, and fat-soluble vitamins (especially vitamin D) deficiencies (36). Therefore, it is necessary to increase the total amount of food in the diet to ensure adequate intake of nutrients and energy because women during special physiologic periods (such as pregnancy), patients and the elderly with limited appetite, and children are particularly vulnerable to energy or malnutrition.

In terms of population growth, infants with a precise vegetarian diet containing milk and dairy products exhibit normal growth and development, but among those following absolute vegetarian diets, heat energy, protein, calcium, iron, zinc, vitamin D, riboflavin, and other B vitamins are inadequate, and thus are prone to a number of nutrient deficiencies. Iron stores get depleted 4–6 months after a baby is born, and the content of iron in milk becomes insufficient; thus, infants >4 months of age must absorb iron from meals, otherwise they will develop iron deficiency anemia. Children who are breastfed for 6 months or longer are prone to rickets when they are fed a vegan diet with minimal vitamin D after weaning. Pre-schoolers, 18 months to 5 years of age, who adopt an absolute vegetarian diet show slow growth and development, and were significantly shorter and weight less than children who eat a balanced diet (37–39). Puberty is the most vigorous period of growth. The requirement for nutrients increases greatly, and puberty is a period of increased sensitivity to nutrient deficiency. If teenagers adopt a vegetarian diet and the type and quantity of food is not designed properly, teenagers will face the risk of a series of nutrient deficiencies, especially calcium, thermal energy, iron, zinc, vitamins A and D, and protein, which will seriously affect growth and development. The requirements for pregnant women and lactating mothers with respect to heat, calcium, vitamins A, C, and D, iron, and folic acid are greatly increased. Malnutrition during pregnancy can also cause intrauterine growth retardation, congenital malformations, and low body weight in the fetus (39, 40). Osteoporosis is the most common degenerative bone metabolic disease among the elderly, especially postmenopausal women, and is an important cause of pathologic fractures in the elderly. People who eat a vegetarian diet tend to be deficient in calcium, iron, vitamin D, and vitamin B12, and have low levels of protein and total fat, which can reduce bone density and increase the risk of fractures (41, 42).

### Healthy bone diet

In recent years, the relationship between dietary patterns and bone health has received increasing attention (43). According to a study of people aged 20–25, five dietary patterns (healthy, traditional, refined, society, rich in nuts and meat products), adhere to nuts and a meat-eating pattern in women is associated with a higher bone mineral density and bone mineral content (44). Another study reported that a diet rich in milk and dairy was better for bone health than other diets (45). Vegetables and fruits in vegetarian diets are rich in minerals (such as calcium, potassium, magnesium, phytocarotene, vitamin, and phytochemicals [such as phytoestrogens]) and many other nutrients that can affect calcium absorption or bone reconstitution (46–48).

The 2015–2020 dietary guidelines for US residents were issued in January 2016 and recommended healthy eating patterns, including more vegetables, fruits, whole grains, low-fat dairy products, nuts, and seafood and less red and processed meats, sugar-sweetened beverages, salt, and refined carbohydrates, and limit cholesterol intake (49).

## Conflicts of interest and funding

The authors have not received any funding or benefits from any industry or elsewhere to conduct this study.
